# Protocol to prepare PARPi-combined LIN28B siRNA nanoparticles and evaluate therapeutic efficacy of malignant ascites

**DOI:** 10.1016/j.xpro.2026.104854

**Published:** 2026-09-26

**Authors:** Yan Fang, Jianfeng Shen

**Affiliations:** 1 Department of Ophthalmology, Ninth People’s Hospital, Shanghai Jiao Tong University School of Medicine, Shanghai 200025, China

**Keywords:** Cancer, Biotechnology and bioengineering, Material sciences

## Abstract

Malignant serous effusion (MSE) is a life-threatening complication of advanced cancers with limited effective treatments. Here, we present a protocol for the preparation of LIN28B siRNA-loaded nanoparticles and their combination with PARP inhibition for therapeutic evaluation in malignant ascites (MA). We describe steps for nanoparticle formulation, establishment of murine ascites models, and *in vivo* administration of therapeutic agents. We then detail procedures for assessing tumor progression, vascular permeability, and endothelial junction integrity to evaluate treatment efficacy and microenvironmental remodeling.

For complete details on the use and execution of this protocol, please refer to Fang et al.^1^

## Highlights

• Steps for preparing LIN28B siRNA-loaded nanoparticles
• Procedures for establishing a murine malignant ascites model
• Instructions for administering nanoparticles and PARP inhibitors *in vivo*
• Methods for assessing tumor burden and vascular permeability

## Abstract (editor highlights)

Publisher’s note: Undertaking any experimental protocol requires adherence to local institutional guidelines for laboratory safety and ethics.

## Graphical abstract

**Figure:**
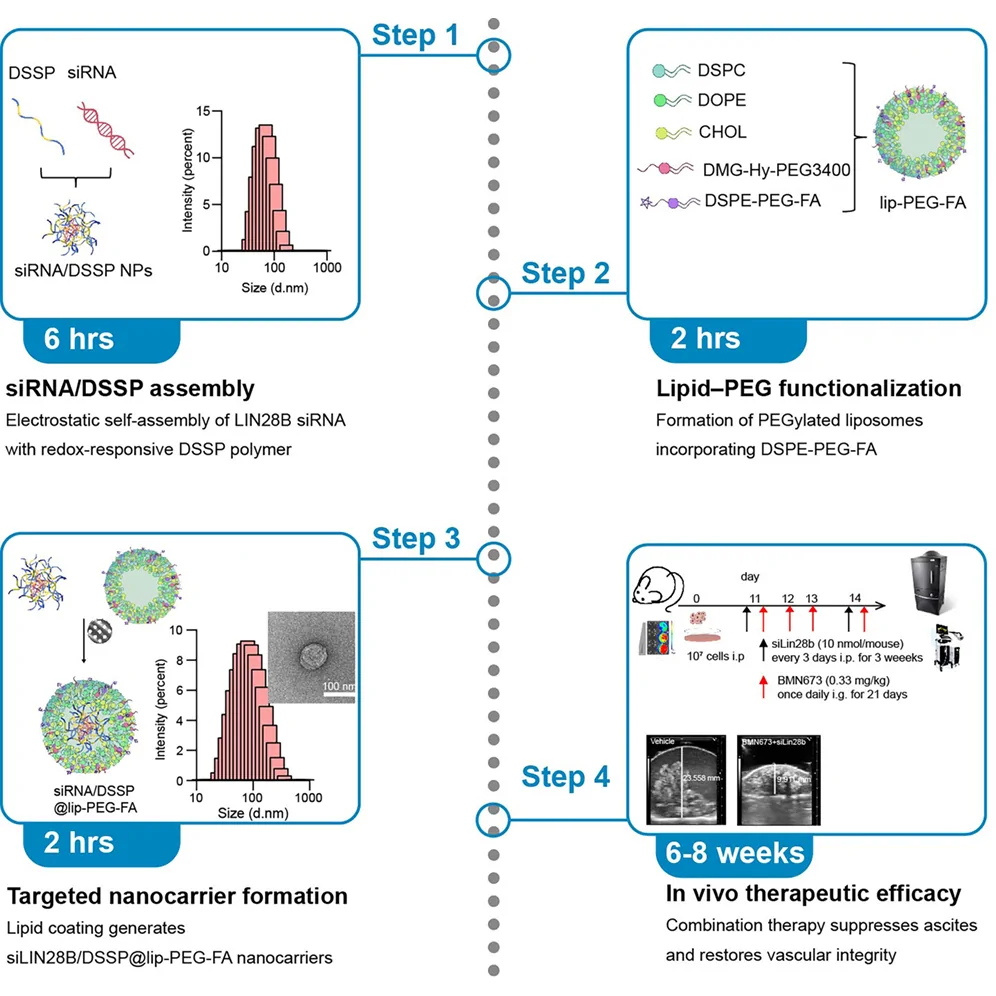

## Before you begin

### Institutional permissions

All animal experiments were conducted in accordance with the institutional ethical guidelines and approved by the Animal Care and Use Committee of Shanghai Jiao Tong University (Approval No. A2023097-003). All procedures were performed in compliance with the approved protocol.

### Background

Malignant ascites (MA), a major subtype of malignant serous effusion (MSE), is a common and life-threatening complication of advanced cancers, particularly ovarian, hepatobiliary, pancreatic, and gastric malignancies.^2^^,^^3^ Clinically, MA is associated with poor prognosis, frequent recurrence, and limited treatment options.^4^^,^^5^^,^^6^ Conventional management strategies—including paracentesis, diuretics, peritoneovenous shunting, and intraperitoneal chemotherapy—primarily provide symptomatic relief but fail to achieve durable therapeutic efficacy and are often accompanied by significant adverse effects.^3^^,^^7^^,^^8^ These limitations highlight the need for mechanistically informed therapeutic strategies.

Recent studies have identified LIN28B as a key regulator involved in tumor progression and treatment resistance, while PARP inhibition has emerged as an effective strategy for targeting DNA damage repair pathways in cancer.^9^^,^^10^^,^^11^^,^^12^ Based on these insights, combined inhibition of PARP and LIN28B was selected as a therapeutic strategy for MA.

The protocol below describes the experimental workflow used to evaluate the therapeutic efficacy of combined PARP inhibition and LIN28B silencing in murine malignant ascites models. The approach includes nanoparticle-based siRNA delivery, *in vivo* treatment administration, and functional assessment of tumor burden, vascular permeability, and endothelial integrity. While optimized for MA models used in our study, this protocol can be adapted to other MSE types (e.g., malignant pleural effusion) and related cancer models.

### Innovation

This protocol establishes a mechanistically guided therapeutic strategy for MA, advancing beyond conventional symptomatic management. A key innovation is the combined targeting of aberrant LIN28B expression and DNA repair vulnerability through the integration of tumor-targeted LIN28B siRNA nanoparticles with the PARP inhibitor BMN673. This approach provides a therapeutic entry point for targeting the LIN28B signaling axis in MA and extends the application of PARP inhibition to this disease context.

In addition, the protocol unifies RNA interference–based nanodelivery with small-molecule inhibition into a single workflow, enabling simultaneous modulation of tumor-intrinsic signaling and genomic instability. This combinatorial design provides a coordinated strategy to enhance therapeutic efficacy.

Importantly, the protocol incorporates *in vivo* evaluation of vascular permeability and endothelial integrity, linking therapeutic response to functional changes in the tumor microenvironment. This adds a mechanistic dimension that is often lacking in conventional MA treatment approaches.

Overall, this workflow provides a modular and adaptable framework for precision therapy in MA, with potential applicability to other malignant serous effusions and related cancer models.

### Preparation 1: Preparation of siRNA/DSSP nanoparticles and construction of lipid-PEG-FA nanocarriers

**Timing: ∼8–10 h**

This section describes the formation of redox-responsive siRNA/DSSP nanoparticles and subsequent lipid–PEG coating to construct tumor-targeted nanocarriers (Figure 1).

**Figure 1:**
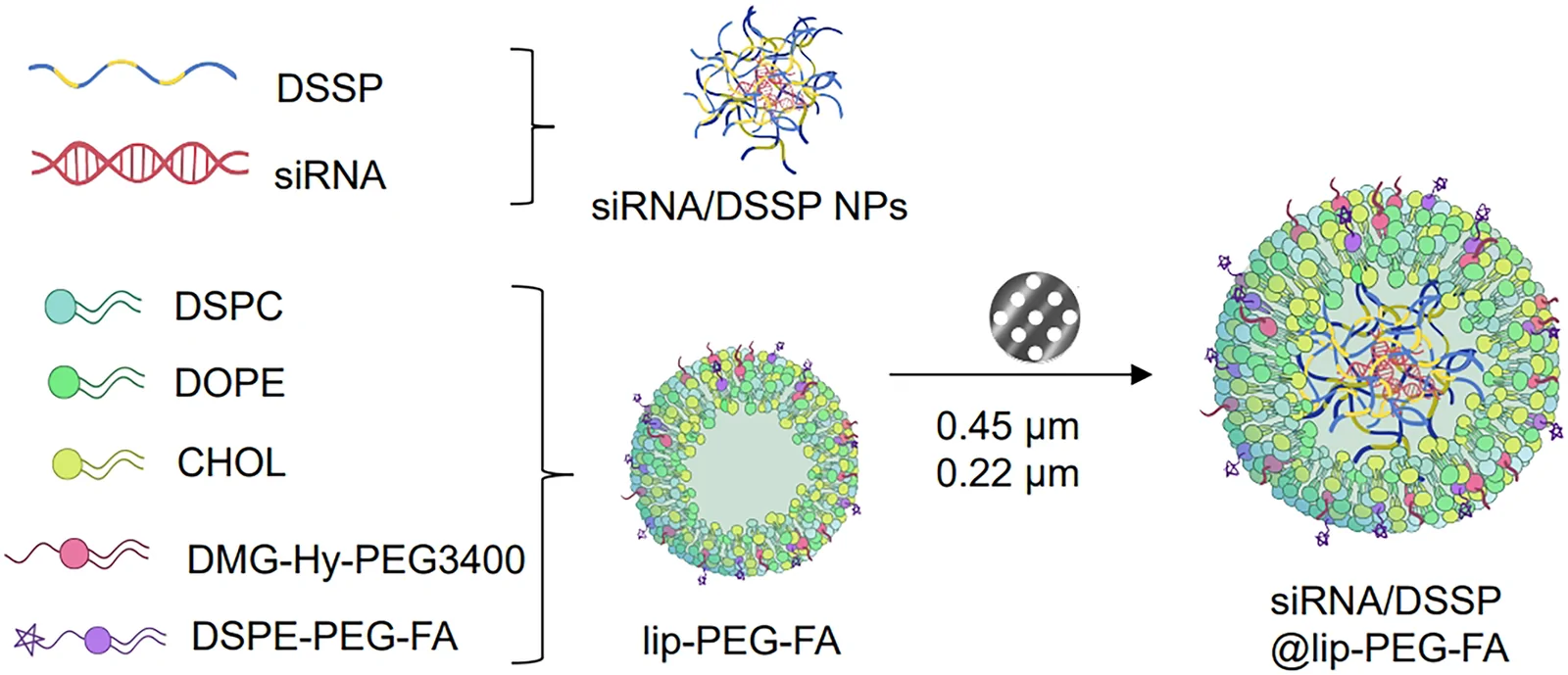
Construction of siRNA/DSSP@lip-PEG-FA nanoparticles siRNA/DSSP nanoparticles were prepared via electrostatic self-assembly between siRNA and DSSP polymer. Lipid vesicles composed of cholesterol, DOPE, and DSPC were generated by thin-film hydration followed by sequential extrusion through 0.45 μm and 0.22 μm membranes. The lipid-coated nanoparticles were further functionalized with DSPE-PEG-FA to obtain tumor-targeted siRNA delivery system.

DSSP is a redox-responsive polymer synthesized in-house according to the previously reported procedure (Fang et al., 2026). DSSP is not commercially available. The detailed synthetic route and physicochemical characterization of DSSP have been reported previously. Researchers interested in reproducing this material may follow the published synthesis protocol or contact the corresponding authors for additional information.

1. Design and preparation of siLIN28B siRNA.
  a. Targeting LIN28B siRNA (siLIN28B) duplexes are designed and synthesized by a commercial vendor.
2. Formation of siRNA/DSSP nanoparticles via electrostatic self-assembly.
  a. Dissolve DSSP polymer in sterile 150 mM NaCl to a final concentration of 0.4 mg/mL under gentle stirring at room temperature.
  b. Prepare siLIN28B or fluorescently labeled siRNA in RNase-free water.
  c. Mix equal volumes of DSSP polymer solution (0.4 mg/mL) and siRNA solution (100 μg/mL) to achieve a DSSP: siRNA mass ratio of 4:1 (mDSSP/msiRNA, w/w). Prepare the nanoparticles in a total reaction volume of 100–200 μL, resulting in a final DSSP concentration of 0.2 mg/mL and a final siRNA concentration of 50 μg/mL.
  d. Incubate the mixture at room temperature for 20–30 min to allow spontaneous nanoparticle self-assembly.
    **CRITICAL:** Avoid vigorous mixing to prevent siRNA degradation.
  e. Continue incubation for an additional 1–2 h under gentle agitation to stabilize nanoparticle formation.
  f. Dilute the nanoparticle suspension 10-fold in filtered ultrapure water (Milli-Q water) immediately before measurement. Measure the hydrodynamic diameter, polydispersity index (PDI), and zeta potential using a Zetasizer Nano (Malvern Panalytical, Malvern, UK). Perform all measurements at 25°C using disposable polystyrene cuvettes for particle size analysis and folded capillary cells for zeta potential measurements. Record at least three independent measurements for each sample and report the results as mean ± SD.
3. Purification of siRNA/DSSP nanoparticles.
  a. Transfer the nanoparticle suspension into dialysis tubing (MWCO: 20 kDa).
  b. Dialyze against deionized water or PBS (pH 7.4) at 4°C.
  c. Replace dialysis buffer every 2–3 h for at least three cycles.
  d. Collect purified nanoparticles for subsequent use.
4. Preparation of blank lipid liposomes.
  a. Cholesterol, DOPE, and DSPC were dissolved in ethanol (1 mL) at an equal molar ratio of 1:1:1 and transferred into a round-bottom flask.
  b. The ethanol solvent was removed by rotary evaporation at 40°C with a rotation speed of 100 rpm under reduced pressure (200 mbar) until a uniform thin lipid film was formed on the inner wall of the round-bottom flask.
  c. Hydrate the lipid film with 10% (w/v) glucose solution at 37°C for 30 min with gentle agitation.
  d. The hydrated lipid suspension was subjected to bathe sonication (40 kHz, 100 W) for 10 min at room temperature to reduce particle size and obtain uniform liposomes.
  e. The blank liposomes were mixed with preformed siLIN28B/DSSP nanoparticles and sequentially extruded through 0.45 μm and 0.22 μm polycarbonate membranes for 20 cycles.
  f. Add DSPE-PEG-FA to the liposome suspension and incubate for 10 min at room temperature with gentle mixing to complete surface functionalization.
5. Construction of siLIN28b/DSSP@lip-PEG-FA nanocarriers (Figure 2).
  a. Mix siRNA/DSSP nanoparticles with blank liposomes to allow adsorption and membrane fusion.
  b. Extrude the mixture through 0.45 μm and 0.22 μm polycarbonate membranes for 20 cycles to obtain uniform particle size.
  c. Collect final lipid–PEG-coated nanoparticles.
  d. Characterize the physicochemical properties of the final nanoparticles by dynamic light scattering (DLS), including hydrodynamic diameter, PDI, and zeta potential.
  e. Determine siRNA encapsulation efficiency using a RiboGreen assay after nanoparticle disruption with a surfactant.
  f. Examine nanoparticle morphology using transmission electron microscopy (TEM).
  g. Confirm siRNA incorporation and protection by agarose gel electrophoresis under free siRNA, nanoparticle-loaded siRNA, and disrupted nanoparticle conditions.

**Figure 2:**
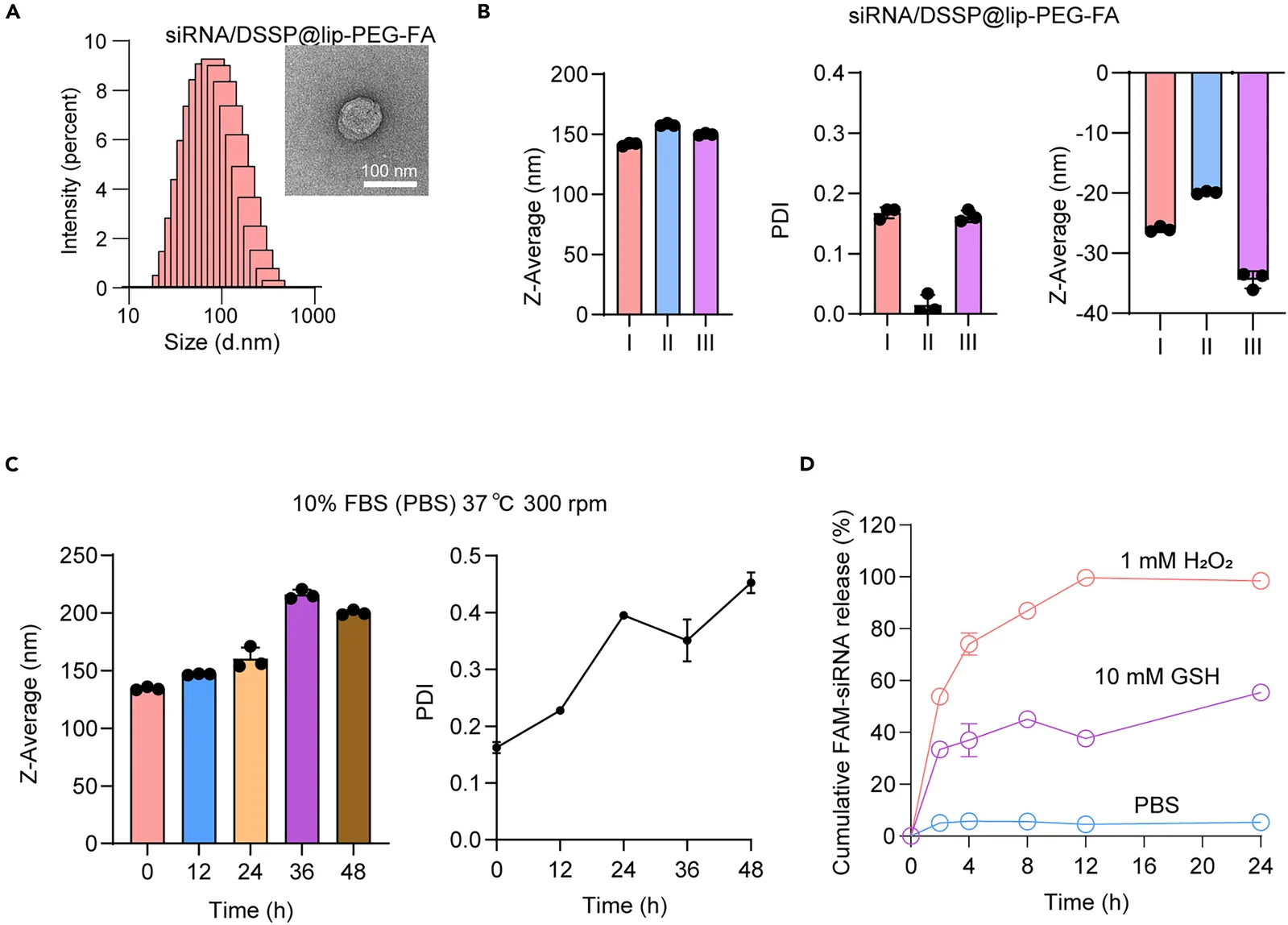
Characterization of siRNA/DSSP@lip-PEG-FA nanoparticles (A) Size distribution of siRNA/DSSP@lip-PEG-FA nanoparticles measured by DLS and representative TEM image. Scale bar, 100 nm. (B) Hydrodynamic diameter (Z-average), PDI, and zeta potential of nanoparticles from three independent preparations (I–III). (C) Stability of nanoparticles in PBS containing 10% fetal bovine serum (FBS) at 37°C under agitation (300 rpm), showing changes in particle size (left) and PDI (right) over time. (D) *In vitro* release profile of FAM-labeled siRNA from nanoparticles under different conditions (PBS, 10 mM GSH, and 1 mM H_2_O_2_), indicating redox-responsive release behavior. Data are presented as mean ± SD (n = 3 biological replicates).
6. Evaluation of cellular uptake and LIN28B knockdown efficiency (Figure 3).
  a. Seed target cells in appropriate culture plates and incubate overnight.
  b. Treat cells with fluorescently labeled siLIN28B/DSSP@lip-PEG-FA nanoparticles to evaluate cellular uptake.
  c. After incubation, wash cells and analyze nanoparticle internalization by confocal laser scanning microscopy (CLSM).
  d. Extract total protein from treated cells.
  e. Evaluate LIN28B silencing efficiency by western blot analysis.

**Figure 3:**
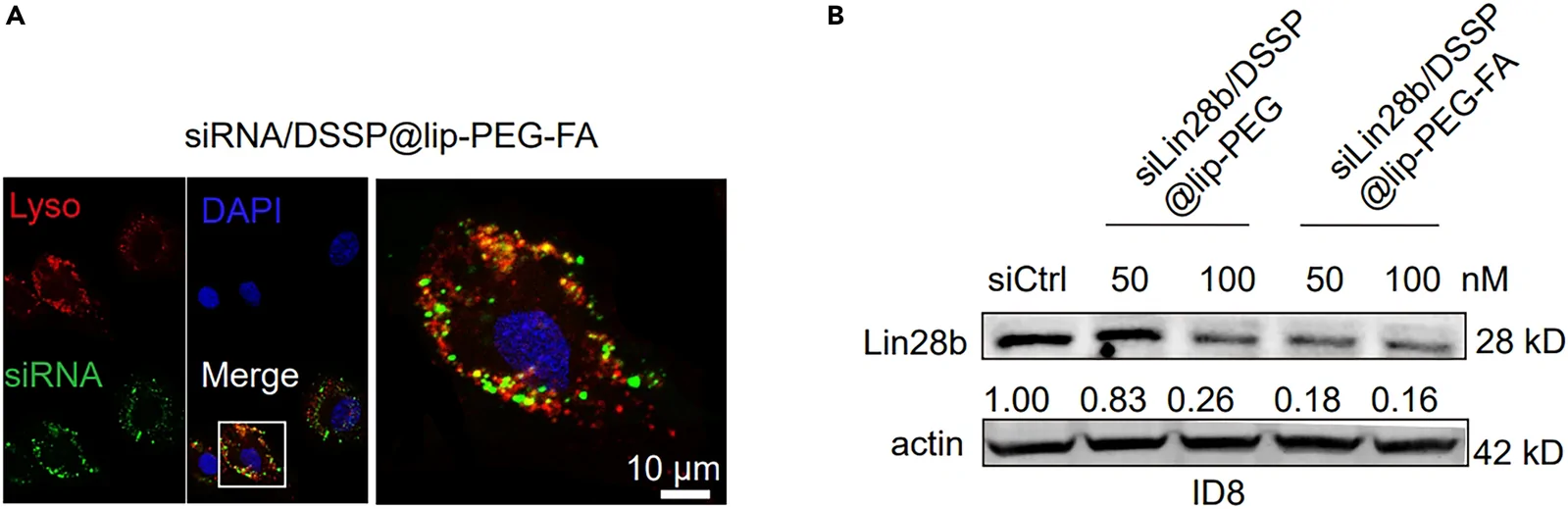
Cellular uptake and gene silencing efficiency of siRNA/DSSP@lip-PEG-FA nanoparticles (A) Confocal fluorescence imaging of intracellular uptake and lysosomal localization of siRNA delivered by siRNA/DSSP@lip-PEG-FA nanoparticles. Lysosomes were stained with LysoTracker (red), nuclei with DAPI (blue), and siRNA was labeled with a fluorescent probe (green). The merged image shows partial colocalization of siRNA with lysosomes, indicating endosomal/lysosomal trafficking following cellular internalization. Scale bar, 10 μm. Scale bar, 10 μm. (B) Western blot analysis of LIN28B protein expression in ID8 cells following treatment with siLin28b/DSSP@lip-PEG or siLin28b/DSSP@lip-PEG-FA nanoparticles at indicated concentrations (50 and 100 nM). β-actin was used as a loading control. Quantification of band intensity (normalized to actin) is shown below each lane, demonstrating enhanced gene silencing efficiency with folate-modified nanoparticles.
7. Storage of nanoparticles.
  a. Store nanoparticles at 4 °C for short-term use (≤1 week).
  b. Avoid freezing, as low temperatures may induce lipid phase transition and aggregation.

**CRITICAL:** Prepare nanoparticles fresh whenever possible for *in vivo* studies.

### Preparation 2: Establishment of luciferase-labeled malignant ascites model and evaluation of combination therapy

**Timing: 6–8 weeks**

**Timing: ∼5–7 days for step 13**

**Timing: ∼3–4 days for step 14**

This section describes the establishment of a luciferase-labeled malignant ascites model and the evaluation of combined treatment with siLIN28B nanoparticles and BMN673.

8. Establishment of malignant ascites model.
  a. Prepare ID8-luc cells with viability >95% and resuspend 1 × 10^7^ cells in 200 μL sterile PBS.
    Inject cells intraperitoneally into female C57BL/6 mice (6–8 weeks old).
  b. Monitor mice daily for ascites development and general health status.
  c. On day 10 post-injection, administer D-luciferin (150 mg/kg, intraperitoneally). After 5–10 min, perform bioluminescence imaging using an IVIS Lumina II system under identical acquisition parameters (exposure time, binning, and field of view) for all groups.
  d. Quantify tumor burden based on luminescence intensity.
9. Treatment grouping and administration.
  a. Perform baseline IVIS imaging on day 10 and randomly assign mice into four groups based on comparable initial tumor burden.
  b. Administer siLIN28B nanoparticles intraperitoneally (10 nmol per mouse) every 3 days.
  c. Administer BMN673 daily by oral gavage (0.33 mg/kg).
  d. For the combination treatment group, administer siLIN28B nanoparticles intraperitoneally every 3 days and BMN673 by oral gavage daily at the same doses and schedules as described above.
  e. Maintain mice under standard housing conditions throughout the treatment period.
  f. Perform all administrations at consistent time points to minimize variability.
10. Monitoring tumor progression and ascites development.
  a. Perform longitudinal IVIS imaging at predefined time points using identical acquisition settings. Quantify total photon flux (photons/s) using IVIS imaging software.
  b. Measure abdominal circumference using calipers.
  c. Quantify ascites accumulation using ultrasound imaging (VEVO LAZR-X).
  d. Define survival endpoint as abdominal circumference exceeding twice that of healthy mice or according to predefined humane endpoints.
11. Sample collection and cytokine analysis.
  a. Euthanize mice at the experimental endpoint.
  b. Centrifuge ascites fluid and serum samples at 3,000 × g for 10 min at 4 °C to remove cellular debris. Collect supernatants and store at −80 °C until analysis.
  c. Perform multiplex cytokine analysis using the Luminex xMAP platform according to the manufacturer’s instructions. Cytokine concentrations are calculated based on standard curves generated from reference standards.
  d. Analyze data using computational pipelines and visualize using Python.
12. Assessment of vascular permeability.
  a. Inject Evans blue dye (30 mg/kg) via tail vein 10 min before sacrifice.
  b. Perfuse mice with PBS to remove intravascular dye.
  c. Harvest abdominal tissues (mesentery and peritoneum), weigh samples, and incubate tissues in formamide at 55°C overnight to extract Evans blue dye.
  d. Measure absorbance at 620 nm using a microplate reader. Normalize Evans blue content to tissue weight and express results as μg dye/g tissue.
13. Whole-mount immunostaining and tissue clearing of mesenterium and peritoneum.
  This procedure describes whole-mount immunostaining and tissue clearing of mesenterium and peritoneum tissues to visualize tumor-associated vascular remodeling, endothelial integrity, and vascular leakage in three dimensions.
  a. Collect mesenterium and peritoneum tissues immediately after euthanasia and gently rinse samples with PBS to remove residual blood.
  b. Fix tissues in 4% paraformaldehyde (PFA) overnight at 4°C.
  c. Wash tissues with PBS and permeabilize/block samples in 10% donkey serum containing 5% Triton X-100 in PBS overnight at 4°C.
  d. Incubate tissues with primary antibodies against CD31 (1:200) and TER-119 (1:100) diluted in antibody incubation buffer (10% donkey serum, 1% BSA, 0.2% Tween-20 in PBS) for 48 h at 4°C.
  e. Wash tissues three times with 0.3% Tween-20/PBS (2 h each).
  f. Incubate tissues with fluorescent secondary antibodies for 48 h at 4°C.
  g. Wash tissues three times and clear samples using Rapiclear 1.49.
  h. Acquire three-dimensional images using spinning disk confocal microscopy.
14. *In vitro* endothelial barrier integrity assay.
  This assay evaluates inflammatory cytokine-induced endothelial junction disruption and the protective effects of siLIN28B/DSSP@lip-PEG-FA and BMN673 treatment by analyzing VE-cadherin expression and junctional continuity in HUVECs.
  a. Seed HUVECs in 12-well plates and culture until cells reach approximately 70%–80% confluence before transfection.
  b. Incubate the transfection mixture with HUVECs for 6 h at 37°C. The final siRNA concentration is 80 pmol per well.
  c. Replace the transfection medium with fresh endothelial growth medium and culture cells for an additional 24 h.
  d. Treat cells with BMN673 (10 nM) or vehicle control for 24 h.
  e. Induce inflammatory endothelial injury by stimulating cells with TNF-α (100 ng/mL) or LPS (1 μg/mL) for 24 h before immunostaining.
  f. Fix cells with 4% paraformaldehyde for 10 min at 4°C, followed by washing with PBS.
  g. Permeabilize and block cells with 10% donkey serum containing 0.5% Triton X-100 in PBS for 1 h at room temperature.
  h. Incubate cells with anti-VE-cadherin primary antibody (1:200 dilution, Cell Signaling Technology) prepared in blocking buffer containing 10% donkey serum, 1% BSA, and 0.2% Tween-20 in PBS overnight at 4°C.
  i. Wash cells three times with 0.3% Tween-20 in PBS (30 min per wash).
  j. Incubate cells with fluorescent secondary antibodies diluted in the same antibody dilution buffer for 1 h at room temperature.
  k. Wash cells three times with PBS containing 0.3% Tween-20 and mount samples using ProLong mounting medium.
  l. Acquire fluorescence images using a confocal microscope with identical acquisition parameters among experimental groups.
  m. Quantify endothelial junction integrity by measuring VE-cadherin-positive area normalized to total nuclear number using ImageJ/Fiji.
15. *In vivo* biocompatibility evaluation.
  a. Collect major organs (heart, liver, spleen, lung, and kidney).
  b. Organs were fixed in 4% PFA, paraffin embedded, sectioned, and stained with H&E according to standard procedures.
  c. Serum biochemical parameters were analyzed using an automated biochemical analyzer according to the manufacturer’s instructions.

## Key resources table

**Table:** 

REAGENT or RESOURCE	SOURCE	IDENTIFIER
**Antibodies**		

Anti-CD31 antibody (1:200)	Thermo Fisher Scientific	Cat#MA3105
Anti-TER-119 antibody (1:100)	BD Biosciences	Cat#557915
Anti-VE-cadherin antibody (1:200)	Cell Signaling Technology	Cat#2500
Anti-mouse Lin28b (1:500)	Proteintech	Cat#11724-1-AP
Anti-human LIN28B (1:1000)	Cell Signaling Technology	Cat#4196S
Anti-β-actin (1:10000)	ABclonal	Cat#AC026
StarBright Blue 520 goat anti-mouse IgG	Bio-Rad	Cat#12005867
Alexa Fluor™ Plus 800 goat anti-rabbit IgG	Invitrogen	Cat#A32735

**Biological samples**		

Mouse ascites fluid	This study	N/A
Mouse serum samples	This study	N/A
Mouse mesenterium and peritoneum tissues	This study	N/A
Major organs (heart, liver, spleen, lung, kidney)	This study	N/A

**Chemicals, peptides, and recombinant proteins**		

DSSP polymer (redox-responsive polymer)	Synthesized in-house according to the previously reported protocol (Fang et al., 2026)	Not commercially available
Cholesterol	Tianjin Xinsi Biochemical Technology Co., Ltd	CAS:57-88-5
DSPE	Ruixi Biological Technology Co., Ltd	CAS:1069-79-0
DOPE (1,2-dioleoyl-sn-glycero-3-phosphoethanolamine)	Ruixi Biological Technology Co., Ltd	CAS:4004-05-1
DMG-Hy-PEG3400	Ruixi Biological Technology Co., Ltd	Cat#R-S0011
DSPE-PEG-FA (folic acid–PEGylated lipid)	Aladdin	Cat#B2224303
Talazoparib (BMN673)	MedChemExpress	CAS:1207456-01-6
Evans blue dye	Sigma-Aldrich	CAS:314-13-6
Triton X-100	Sigma-Aldrich	CAS:9036-19-5
Donkey serum	Absin	Cat#935
TNFα	R&D Systems	Cat#210-TA-100
Lipopolysaccharide (LPS)	Sigma-Aldrich	Cat#L2630
ProLong mounting medium	Invitrogen	Cat#P36961
BSA	Sangon Biotech (Shanghai) Co., Ltd	Cat#A600332
Tween-20	Sangon Biotech (Shanghai) Co., Ltd	Cat#A600560
Sucrose	Aladdin	CAS:57-50-1
D-luciferin	Genomeditech(Shanghai) CO., Ltd	CAS:103404-75-7
Glucose	Aladdin	CAS:14431-43-7
Dulbecco’s Modified Eagle’s Medium (DMEM)	Gibco	Cat#11965092

**Critical commercial assays**		

Luminex xMAP multiplex cytokine assay	Luminex	Multiplex panel
H&E staining kit	Beyotime	Cat#C0105
Serum biochemical analysis kits	Nanjing Jiancheng Bioengineering Institute	Various

**Experimental models: Cell lines**		

ID8 cell line	Gift	N/A
HUVEC (human umbilical vein endothelial cells)	PromoCell	Cat#C-12208

**Experimental models: Organisms/strains**		

C57BL/6 mice (female, 6–8 weeks old)	Beijing Vital River Laboratory Animal Technology Co., Ltd	Strain: C57BL/6; License No. SCXK (Beijing) XXXX

**Oligonucleotides**		

silin28b mouse (sense):	This study	5′-GGAUGUAUUUGUACACCAAtt-3′
silin28b mouse (antisense):	This study	5′-UUGGUGUACAAAUACAUCCtt-3′
siLIN28B Human(sense)	This study	5′-CCUCCACGAAGUCAUCUAUTT-3′
siLIN28B Human(antisense)	This study	5′-AUAGAUGACUUCGUGGAGGTT-3′
Fluorescently labeled siRNA	GenePharma	Custom
Primer: Human LIN28B_forward:	This study	AAGAAGACCCAAAGGGAAGACAC
Primer: Human LIN28B_reverse:	This study	ACACCATGGGGAAGGTGAAG

**Software and algorithms**		

Python	Python Software Foundation	https://www.python.org
Matplotlib	Matplotlib	https://matplotlib.org
VEVO LAZR-X imaging software	FUJIFILM VisualSonics	VEVO LAZR-X
IVIS imaging software	PerkinElmer	IVIS Lumina II

**Other**		

Dialysis tubing (MWCO: 20 KDa)	Thermo Fisher Scientific	Thermo Scientific™ Slide-A-Lyzer™ MINI Dialysis Device, 20K MWCO
Polycarbonate extrusion membranes	Millipore	0.22 μm/0.45 μm
IVIS Lumina II imaging system	PerkinElmer	Imaging system
VEVO LAZR-X ultrasound imaging system	FUJIFILM VisualSonics	Imaging system
Dynamic light scattering (DLS) instrument	Malvern Panalytical	Zetasizer Nano
Rapiclear tissue clearing reagent	SUNJin Lab	Cat#RC149001
Spinning disk confocal microscope	Andor	Dragonfly

## Materials and equipment

### Nanoparticle characterization and preparation

• Dynamic light scattering instrument: Zetasizer Nano (Malvern Panalytical).
• Liposome extrusion system: Avanti mini-extruder (Avanti Polar Lipids) with polycarbonate membranes (0.45 μm and 0.22 μm).
• Dialysis device (MWCO: 20,000 Da): Thermo Scientific™ Slide-A-Lyzer™ MINI Dialysis Device (Thermo Fisher Scientific).
• UV–visible spectrophotometer for nucleic acid quantification.

### *In vivo* imaging systems

• IVIS Lumina II imaging system (PerkinElmer).
• VEVO LAZR-X ultrasound imaging system (FUJIFILM VisualSonics).

### Microscopy and imaging

• Spinning disk confocal microscope (Dragonfly, Andor).
• Objectives: 20× and 40× (oil immersion).
• Laser lines: 488 nm, 561 nm, 640 nm.

### Molecular and cellular analysis

• Flow cytometer (BD FACSCanto II, BD Biosciences).
• Image analysis software: ImageJ/Fiji (NIH).
• Cytokine analysis platform: Luminex xMAP system with dedicated analysis software.
• Data visualization: Python (v3.x) with Matplotlib.

### Histology and tissue processing

• Cryostat embedding system for OCT processing.
• H&E staining kit (commercial standard kit).
• Tissue clearing reagent: Rapiclear (SunJin Lab).

### Endothelial permeability and biochemical assays

• Microplate reader for Evans blue absorbance measurement.
• Automated biochemical analyzer for serum biochemistry assays.

### Recipes (important)

**Table:** Blocking buffer for immunostaining

Component	Final concentration
Donkey serum	5% (v/v)
PBS	—

Store at 4 °C for up to 1 week.

**Table:** Permeabilization buffer

Component	Final concentration
TritonX-100	0.2% (v/v)
PBS	—

Store at 4 °C for up to 1 week.

**Table:** Lipid film hydration solution

Component’	Final concentration
Glucose	10% (v/v)

Prepare fresh or store at room temperature for ≤24 h.

**CRITICAL:** All buffers should be prepared using RNase-free water when handling siRNA-related experiments.

## Step-by-step method details

### Establishment and baseline assessment of the malignant ascites model

**Timing: 10 days**

This step generates a luciferase-labeled malignant ascites mouse model for longitudinal tumor monitoring.

1. Inject 1 × 10^7^ ID8-luc cells suspended in sterile PBS (200 μL) intraperitoneally into female C57BL/6 mice (6–8 weeks old).
2. Monitor mice daily for body weight, abdominal distension, and overall health status.
3. Perform baseline bioluminescence imaging on day 10 post-injection, which was selected based on preliminary optimization experiments showing that bioluminescent signals were not consistently detectable in all mice at day 7, whereas stable and detectable luciferase signals were observed in the majority of mice by day 10. This time point was therefore chosen to ensure reliable tumor establishment and minimize variability during treatment grouping.
  a. Inject D-luciferin intraperitoneally (150 mg/kg).
  b. Anesthetize mice using isoflurane.
  c. Acquire images using the IVIS Lumina II system.

**CRITICAL:** Ensure consistent timing between luciferin injection and imaging (typically 10–15 min) to minimize signal variability.

### Treatment design and administration

**Timing: 3–5 weeks**

This step evaluates the therapeutic effects of siLIN28B nanoparticles combined with PARP inhibition.

4. Randomly assign mice into four groups (n≥ 5 per group):
  a. PBS control.
  b. siLIN28B nanoparticles.
  c. BMN673.
  d. Combination treatment.
5. Administer siLIN28B nanoparticles (10 nmol per mouse, intraperitoneally) every 3 days.
6. Administer BMN673 (0.33 mg/kg, oral gavage) daily.
7. Continue treatment for the designated experimental period (typically 3–4 weeks).

**CRITICAL:** Maintain consistent dosing time each day to reduce circadian variability.

### Tumor progression and ascites monitoring

**Timing: Throughout treatment period**

This step tracks tumor burden and ascites development.

8. Perform longitudinal *in vivo* bioluminescence imaging at defined time points.
9. Measure abdominal circumference using calipers.
10. Monitor ascites accumulation using ultrasound imaging (VEVO LAZR-X).
11. Define survival endpoint as abdominal circumference exceeding twice that of healthy controls or according to humane endpoint criteria.

***Note:*** Use identical imaging settings across all groups for quantitative comparison.

### Sample collection and cytokine profiling

**Timing: 1 day**

This step enables systemic immune and inflammatory analysis.

12. Euthanize mice at the experimental endpoint following institutional guidelines.
13. Collect ascites fluid and blood via cardiac puncture.
14. Centrifuge samples at 3,000 × g for 10 min at 4 °C to obtain supernatants.
15. Perform multiplex cytokine analysis using the Luminex xMAP platform according to the manufacturer’s instructions.
16. Process and visualize cytokine data using Python and Matplotlib.

### Functional assays of vascular permeability and tissue remodeling

**Timing: 1–2 days**

This step evaluates vascular leakage and microenvironment remodeling.

17. Inject Evans blue dye (30 mg/kg in saline) via tail vein 10 min before sacrifice.
18. Perfuse mice with PBS (5–10 mL) via cardiac perfusion to remove circulating dye.
19. Harvest major organs and peritoneal tissues.
20. Quantify Evans blue extravasation using spectrophotometric analysis.
21. Fix mesenterium and peritoneum tissues in 4% paraformaldehyde at 4 °C overnight.
22. Perform immunofluorescence staining:
  a. Incubate with primary antibodies (CD31, TER-119).
  b. Incubate with corresponding secondary antibodies.
  c. Clear tissues using Rapiclear.
23. Acquire images using a spinning disk confocal microscope.

**CRITICAL:** Avoid over-perfusion, which may reduce detectable Evans blue signal.

### *In vitro* endothelial integrity and systemic toxicity evaluation

**Timing: 2–3 days**

This step assesses endothelial barrier function and treatment safety.

24. Seed HUVECs in culture plates and allow attachment overnight.
25. Transfect HUVECs with siLIN28B or control siRNA.
26. Treat cells with BMN673 for 24 h.
27. Stimulate cells with TNFα (100 ng/mL) or LPS (1 μg/mL) for 24 h.
28. Fix cells with 4% paraformaldehyde and stain for VE-cadherin.
29. Acquire fluorescence images and quantify junction integrity using ImageJ.
30. Collect major organs (heart, liver, spleen, lung, kidney) for histological analysis.
31. Perform H&E staining to assess tissue toxicity.
32. Analyze serum biochemical markers (ALT, AST, BUN, CREA, UA, TBIL) using standard kits.

**CRITICAL:** Use early-passage HUVECs (≤ passage 6) to ensure reproducible endothelial responses.

## Expected outcomes

Implementation of this protocol is expected to establish a robust and reproducible murine model of MA, characterized by progressive abdominal distension, measurable accumulation of ascitic fluid, and strong bioluminescence signals following intraperitoneal implantation of ID8-luc cells. In control (vehicle-treated) mice, tumor progression is typically associated with increased ascites formation, enhanced vascular permeability, and reduced overall survival.

Following successful preparation, siRNA/DSSP@lip-PEG-FA nanoparticles are expected to exhibit a hydrodynamic diameter of approximately 120–180 nm, a PDI below 0.30, and a slightly negative to near-neutral zeta potential (approximately −10 to −25 mV) after lipid–PEG coating. These characteristics indicate successful nanoparticle assembly, homogeneous size distribution, and sufficient colloidal stability for subsequent biological applications (Figure 2).

Treatment with either siLIN28B/DSSP@lip-PEG-FA or BMN673 alone is expected to partially attenuate tumor progression, as evidenced by decreased bioluminescence intensity, delayed ascites accumulation, and modest improvement in survival. In contrast, combination treatment is expected to produce a more pronounced therapeutic effect, including significant reduction in tumor burden, decreased ascitic fluid volume, and prolonged survival compared to monotherapy or control groups (Figure 4).

**Figure 4:**
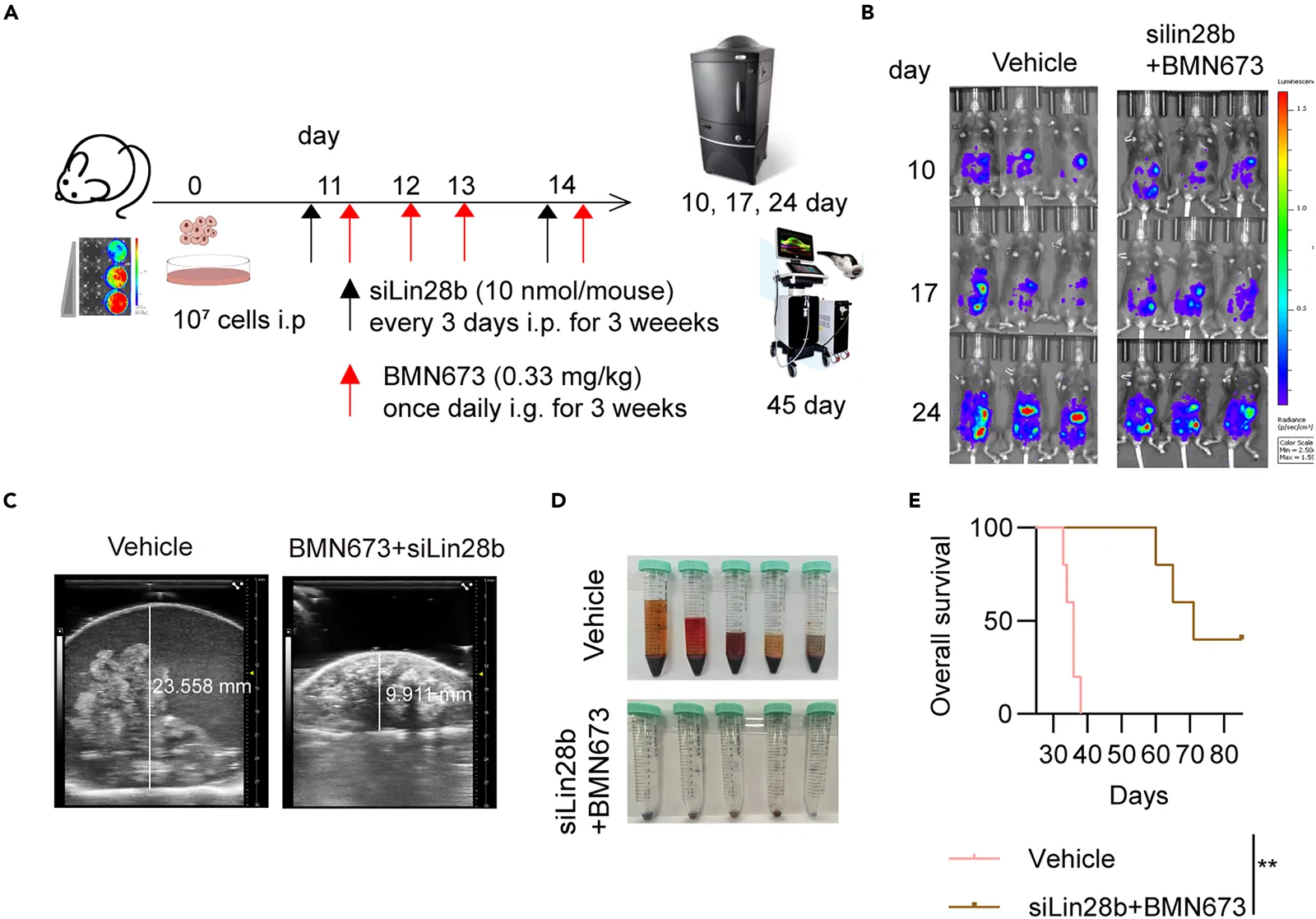
Therapeutic evaluation of siLIN28b/DSSP@lip-PEG-FA combined with BMN673 in a malignant ascites model (A) Schematic illustration of the experimental design and treatment schedule. Female C57BL/6 mice were intraperitoneally injected with 1×10^7^ ID8-luc cells on day 0 to establish the malignant ascites model. After baseline bioluminescence imaging, mice were randomly assigned to the indicated treatment groups (n = 5 mice per group). siLIN28B/DSSP@lip-PEG-FA nanoparticles were administered intraperitoneally every 3 days for 3 weeks, and BMN673 was administered daily by oral gavage for 21 days. Tumor progression was longitudinally monitored by IVIS imaging on days 10, 17, and 24 after tumor inoculation, and mice were followed until the predefined experimental endpoint (day 45). (B) Representative longitudinal IVIS bioluminescence images showing tumor progression in vehicle-treated and siLIN28B/DSSP@lip-PEG-FA + BMN673 combination-treated mice at the indicated time points. The same animals were imaged repeatedly throughout the experiment using identical acquisition settings. (C) Representative ultrasound images of abdominal regions showing differences in ascites accumulation among treatment groups at the experimental endpoint. (D) Representative images of malignant ascites fluid collected from mice in different treatment groups at the experimental endpoint. (E) Kaplan–Meier survival curves of mice receiving different treatments (n = 5 per group). Survival distributions were compared using the log-rank (Mantel–Cox) test.

At the functional level, successful vascular permeability assessment using Evans blue assay is expected to demonstrate reduced extravasation of Evans blue dye in treated groups, particularly in the combination treatment group, indicating attenuation of tumor-associated vascular leakage. Immunofluorescence analysis of mesenteric and peritoneal tissues is expected to reveal improved endothelial organization, with more continuous CD31-positive vascular structures and reduced abnormal vascular disruption compared with vehicle-treated controls (Figure 5).

**Figure 5:**
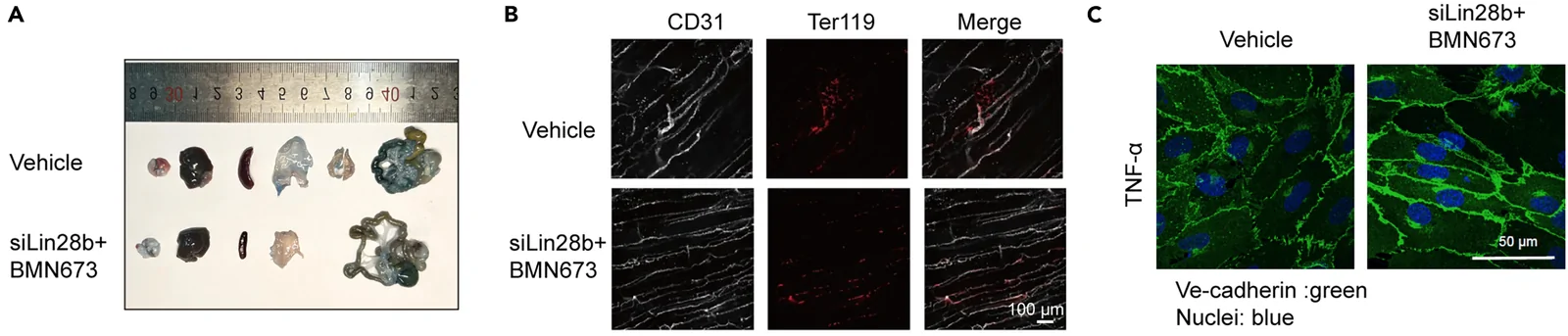
Representative evaluation of vascular permeability and endothelial integrity following therapeutic intervention in the malignant ascites model (A) Representative images of major organs and peritoneal tissues collected from vehicle-treated and siLIN28B/DSSP@lip-PEG-FA plus BMN673-treated mice at the experimental endpoint. Reduced ascites accumulation and tissue abnormalities were observed in the combination treatment group. (B) Representative immunofluorescence staining of mesenteric tissues for endothelial marker CD31 and erythrocyte marker TER-119. Compared with vehicle-treated mice, combination treatment resulted in improved vascular organization, characterized by more continuous CD31-positive endothelial structures and reduced TER-119-positive vascular leakage signals. Scale bar, 100 μm. (C) Representative immunofluorescence images of VE-cadherin staining in HUVECs under indicated treatment conditions. Combination treatment preserved endothelial junction integrity, as indicated by enhanced membrane-localized VE-cadherin distribution and increased junction continuity compared with control conditions. Nuclei were counterstained with DAPI. Scale bar, 50 μm.

Furthermore, *in vitro* endothelial integrity assays using HUVECs are expected to show that inflammatory stimulation (e.g., TNFα treatment) induces endothelial junction disruption, whereas siLIN28B/DSSP@lip-PEG-FA combined with BMN673 treatment preserves endothelial barrier integrity. This effect can be evaluated by VE-cadherin staining, where successful treatment is indicated by enhanced membrane-localized VE-cadherin distribution and increased junction continuity compared with inflammatory controls (Figure 5). The combination treatment exhibited a favorable safety profile, as evidenced by both biochemical parameters and H&E staining, with no apparent signs of systemic or tissue toxicity (Figure 6).

**Figure 6:**
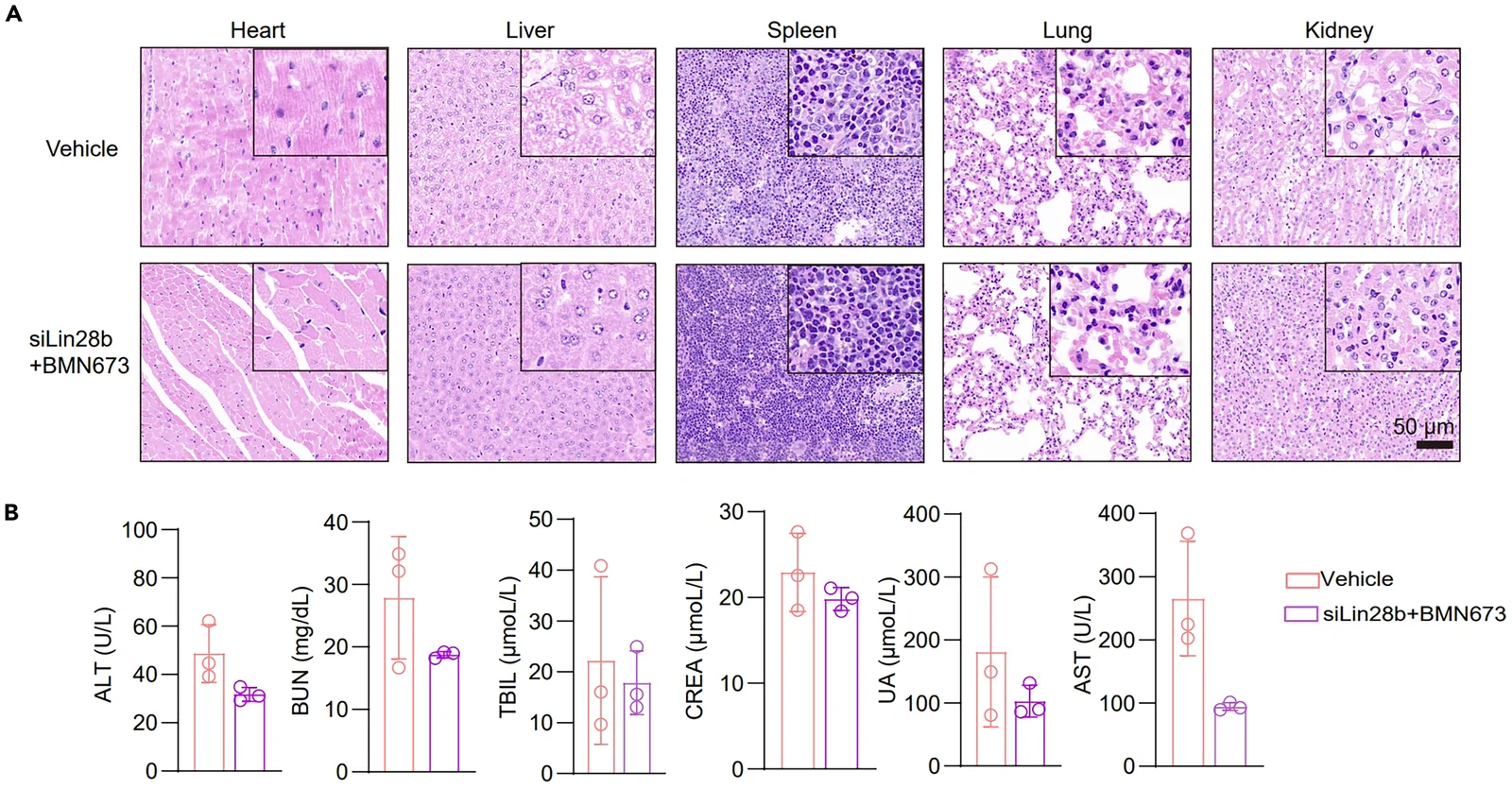
*In vivo* biosafety evaluation of siLIN28b/DSSP@lip-PEG-FA combined with BMN673 (A) Representative hematoxylin and eosin (H&E) staining images of major organs, including heart, liver, spleen, lung, and kidney, collected from mice treated with vehicle or siLIN28b/DSSP@lip-PEG-FA in combination with BMN673. Insets show higher-magnification views of the corresponding regions. No obvious histopathological abnormalities or tissue damage were observed in the treatment group compared with the control. Scale bar, 50 μm. (B) Serum biochemical analysis of liver and kidney function, including alanine aminotransferase (ALT), blood urea nitrogen (BUN), total bilirubin (TBIL), creatinine (CREA), uric acid (UA), and aspartate aminotransferase (AST). Data are presented as mean ± SD (n = 3). No significant differences were observed between groups, indicating minimal systemic toxicity.

Together, these results provide measurable criteria for successful implementation of the protocol, including nanoparticle quality control, tumor suppression, reduced ascites formation, improved vascular permeability, and restoration of endothelial barrier function.

## Quantification and statistical analysis

All quantitative data are presented as mean ± standard deviation (SD), unless otherwise specified. Statistical analyses and data visualization are performed using GraphPad Prism (version 9.0) and Python (version 3.x) with Matplotlib.

For longitudinal imaging analyses, including serial IVIS bioluminescence imaging, tumor burden was monitored repeatedly in the same animals at predefined time points. Comparisons among treatment groups over time were performed using two-way ANOVA with repeated measures followed by appropriate post hoc tests. For single-time-point measurements, comparisons between two groups were performed using unpaired two-tailed Student’s *t* test, while multiple-group comparisons were analyzed using one-way ANOVA followed by Tukey’s multiple comparisons test.

Survival curves were analyzed using the Kaplan–Meier method and compared using the log-rank (Mantel–Cox) test.

For IVIS and ultrasound analyses, identical acquisition parameters were applied throughout all imaging sessions. Quantification was performed using consistent thresholding criteria with ImageJ/Fiji or manufacturer-provided software.

Animals were randomly assigned to experimental groups after baseline imaging to minimize differences in initial tumor burden. The sample size (n) for each experiment is indicated in the corresponding figure legends. Animals or samples were excluded only based on predefined technical criteria, including unsuccessful model establishment, imaging failure, or sample loss during processing. Image acquisition and quantitative analyses were performed blinded to treatment groups whenever applicable.

Statistical significance is defined as P ≥ 0.05 (not significant, ns), P < 0.05 (∗), P < 0.01 (∗∗), and P < 0.001 (∗∗∗).

## Limitations

Despite its robustness and reproducibility, this protocol has several limitations that should be considered when interpreting the results.

First, the ID8 intraperitoneal model, although widely used to study ovarian cancer–associated ascites, does not fully recapitulate the genetic heterogeneity and clinical complexity of human malignant ascites. Therefore, caution should be exercised when extrapolating these findings to patient settings or other tumor types.

Second, nanoparticle formulation, particularly lipid coating via extrusion, may exhibit batch-to-batch variability in particle size distribution, surface properties, and siRNA loading efficiency, which could influence biological performance and treatment outcomes.

Third, the pharmacokinetics and biodistribution of siLIN28B/DSSP@lip-PEG-FA are not comprehensively characterized within this protocol. As a result, interpretation of dose–response relationships and tissue-specific delivery efficiency may be limited.

Fourth, immune characterization in this protocol is primarily based on cytokine profiling and selected histological markers, which may not fully capture the complexity and heterogeneity of the tumor immune microenvironment. Higher-resolution approaches, such as single-cell sequencing, may provide additional insights.

Finally, the combination of RNA interference–based therapy and small-molecule inhibition requires careful coordination of dosing schedules and administration routes. Variations in timing or dosing conditions across experiments may introduce variability and should be carefully controlled.

## Troubleshooting

Below are common issues encountered during nanoparticle preparation, lipid coating, and *in vivo* evaluation, along with recommended solutions. Each issue is linked to the corresponding step(s) in the protocol.

### Problem 1

Incomplete dissolution of DSSP polymer (Preparation 1, Step 2).

DSSP does not fully dissolve, resulting in turbidity or visible particulates.

### Possible solutions

• Extend stirring time at room temperature or apply mild heating (≤37 °C).
• Prepare DSSP in 150 mM NaCl to maintain appropriate ionic strength.
• Avoid excessive heating to prevent polymer degradation.
• Apply brief bath sonication to facilitate dissolution.
• Filter the solution through a 0.45 μm membrane prior to siRNA mixing if particulates persist.

### Problem 2

Low siRNA loading efficiency or unstable nanoparticle formation (Preparation 1, Steps 2–3).

Nanoparticles exhibit low encapsulation efficiency or poor stability based on DLS measurements.

### Possible solutions

• Optimize the siRNA: DSSP mass ratio within tested range (±10–20%).
• Perform gentle mixing and avoid vigorous vortexing.
• Use RNase-free reagents and confirm siRNA integrity.
• Prepare siRNA solutions freshly prior to use.

### Problem 3

Nanoparticle aggregation or high polydispersity after dialysis (Preparation 1, Step 4).

Particles exhibit aggregation or broad size distribution following purification.

### Possible solutions

• Perform gradual buffer exchange to minimize osmotic stress.
• Maintain dialysis at 4 °C throughout the process.
• Introduce low ionic strength buffer (e.g., 5–10 mM NaCl) if necessary.
• Avoid excessive dialysis duration that may destabilize nanoparticles.
• If the measured particle size is substantially larger than expected (>250 nm) or the PDI exceeds 0.30, optimize the siRNA/DSSP mass ratio, verify complete lipid film hydration, increase extrusion cycles, or prepare a fresh nanoparticle formulation. If the zeta potential deviates markedly from the expected range, confirm buffer composition, sample dilution, and lipid composition before repeating the measurement.

### Problem 4

Inefficient lipid coating during extrusion (Preparation 1, Step 6).

Nanoparticles show non-uniform size distribution or incomplete lipid coating.

### Possible solutions

• Ensure complete removal of organic solvent during lipid film preparation (Step 5).
• Increase extrusion cycles (e.g., from 20 to 25 cycles).
• Pre-equilibrate liposomes and nanoparticles at room temperature before mixing.
• Verify the integrity of extrusion membranes.

### Problem 5

Low DSPE-PEG-FA functionalization efficiency (Preparation 1, Step 6).

Targeting ligand incorporation is insufficient, resulting in reduced cellular uptake.

### Possible solutions

• Extend incubation time during functionalization (e.g., 10–20 min).
• Ensure homogeneous mixing of lipid components.
• Avoid performing functionalization at low temperatures (<20 °C).
• Use fresh DSPE-PEG-FA and avoid repeated freeze–thaw cycles.

### Problem 6

High variability in tumor burden or ascites formation (Preparation 2).

Inconsistent disease progression among mice.

### Possible solutions

• Ensure high cell viability (>95%) before injection.
• Standardize injection volume and intraperitoneal injection technique.
• Randomize animals prior to treatment allocation.
• Exclude animals with abnormal baseline signals during initial imaging.

### Problem 7

Weak IVIS or ultrasound signal (Preparation 2, Step 3).

Low signal intensity or poor visualization of ascites.

### Possible solutions

• Confirm luciferase expression in ID8-luc cells prior to implantation.
• Optimize imaging parameters (e.g., exposure time, filter settings).
• Maintain consistent anesthesia conditions during imaging.
• Ensure proper ultrasound probe positioning and coupling.

### Problem 8

High background in immunofluorescence staining (Preparation 2, Step 6).

Non-specific signal reduces image quality.

### Possible solutions

• Increase blocking duration or serum concentration.
• Extend washing steps (≥3 × 30 min).
• Reduce primary antibody concentration if necessary.
• Ensure complete tissue clearing using Rapiclear before imaging.

### Problem 9

Abnormal serum biochemistry in control groups (Preparation 2, Step 8).

Unexpected elevation of toxicity-related biomarkers (e.g., ALT, AST).

### Possible solutions

• Confirm sterility and endotoxin-free status of formulations.
• Avoid repeated freeze–thaw cycles.
• Verify dosing accuracy and administration route.
• Use freshly prepared formulations.

### Problem 10

Weak cytokine detection in Luminex assay (Preparation 2, Step 4).

Low signal intensity or inconsistent multiplex readouts.

### Possible solutions

• Ensure thorough resuspension of beads before use.
• Extend incubation time for low-abundance targets.
• Minimize bead loss during washing steps.
• Optimize sample dilution to avoid signal loss.

## Resource availability

### Lead contact

Further information and requests for resources and reagents should be directed to and will be fulfilled by the lead contact, Jianfeng Shen (jfshen@shsmu.edu.cn).

### Technical contact

Technical questions on executing this protocol should be directed to and will be answered by the technical contact, Yan Fang (yanyan2021fang@sjtu.edu.cn).

### Materials availability

This study did not generate new unique reagents. The siLIN28B sequences and DSSP-based nanoparticle formulations used in this study are described in detail in the associated publication (Fang et al., Advanced Science, 2026) and can be reproduced following the protocol.

### Data and code availability

• The published article (Fang et al., Advanced Science, 2026) includes all datasets generated or analyzed during this study.
• Additional data supporting the findings of this study are available from the corresponding author upon reasonable request.
• This study did not generate new custom code.

## Acknowledgments

We thank the Center for High Performance Computing at Shanghai Jiao Tong University for technical support. J.S. was supported by the General Program of the 10.13039/100014718National Natural Science Foundation of China (no. 82472752).

## Author contributions

Y.F. and J.S. conceptualized the study. Y.F. designed and conducted the experiments. Y.F. developed and implemented the method. Y.F. and J.S. wrote the manuscript. J.S. provided supervision and funding.

## Declaration of interests

Y.F and J.S. are authors of a patent (publication number 202510447248.0) that relates to the content of this manuscript.
